# Chicken Cathelicidins Display Antimicrobial Activity against Multiresistant Bacteria without Inducing Strong Resistance

**DOI:** 10.1371/journal.pone.0061964

**Published:** 2013-04-22

**Authors:** Edwin J. A. Veldhuizen, Ellen C. Brouwer, Viktoria A. F. Schneider, Ad C. Fluit

**Affiliations:** 1 Department of Infectious Diseases and Immunology, Division of Molecular Host Defence, Faculty of Veterinary Medicine, Utrecht University, Utrecht, The Netherlands; 2 Department of Medical Microbiology, University Medical Center Utrecht, Utrecht, The Netherlands; Institut National de la Recherche Agronomique, France

## Abstract

The increased prevalence of multidrug-resistant (MDR) bacteria in combination with the relatively limited development of new antibiotics presents a serious threat to public health. In chicken, especially Extended-Spectrum ß-Lactamase (ESBL) carrying Enterobacteriaceae are often asymptomatically present but can infect humans. Due to their broad range antimicrobial activity cathelicidins and other host defence peptides, are considered to be an attractive alternative to conventional antibiotics. In this study, the antimicrobial activity of three chicken cathelicidins against a broad array of multidrug resistant bacteria was determined. All three peptides showed high antibacterial activity independent of the presence of MDR characteristics. Induction experiments using *S. aureus* and *K. pneumoniae* showed that although an increase in resistance was initially observed, susceptibility towards chicken cathelicidins remained high and no major resistance was developed. The combined results underline the potential of chicken cathelicidins as a new alternative to antibiotics.

## Introduction

The world-wide increase in antibiotic resistance has severely reduced the current treatment options for infectious diseases. This issue is particularly serious in the case of Extended-Spectrum ß-Lactamase (ESBL) carrying Enterobacteriaceae. ESBLs confer resistance to third-generation cephalosporins, a class of antibiotics that is often used for empiric therapy. The growing levels of third-generation cephalosporin resistance leave only carbapenems as reliable treatment option, however, resistance against these antibiotics is also increasingly reported [1]. Similarly, methicillin-resistant *Staphylococcus aureus* (MRSA) remain a problem with rising numbers of community-acquired MRSA and the global spread of livestock-associated MRSA, in particular ST398 [2], [3]. Alternative treatment options are urgently required to address the dangers posed by these drug-resistant pathogens. Cathelicidins are a class of antimicrobial peptides that may provide this alternative. Chicken cathelicidins are particularly interesting in this respect, considering the fact that the chicken is a major non-symptomatic carrier of multiresistant bacteria.

Cathelicidins are Host Defence Peptides (HDP) that play an important role in the innate immune system. They exhibit broad range antimicrobial activity against both Gram-negative and Gram-positive bacteria, as well as against fungi and parasites. To date, most study has focused on the antibacterial mode of action of these peptides. So-called lytic peptides, such as the human cathelicidin LL-37, bind to bacterial membranes and either form pores or lead to destabilization of the membrane, eventually leading to lysis of the bacteria. Other cathelicidins, such as porcine PR-39 cross the bacterial membrane to access intracellular targets leading to inhibition of protein and DNA synthesis [4], [5]. In addition to antimicrobial activity, many HDPs possess immunomodulatory activities including lipopolysaccharide (LPS) binding, induction of cytokine production and chemotaxis [6]. This multiplicity of functions of the HDPs adds to their potential for use as alternative for antibiotics.

In chicken, four cathelicidins (CATH-1-3 and CATH-B1) have been described [7]–[9]. The first three cathelicidins have shown potent broad spectrum antibacterial activity in vitro [8], [10], [11], while CATH-B1 has only been tested against a limited number of bacterial strains. The mature forms of CATH-1 and CATH-3 share a high sequence homology (>70%), and are thought to be the result of gene duplication [8]. However, no apparent homology is present between CATH-1/-3 with CATH-2 and CATH-B1 (Table 1). Structurally, CATH-1 and CATH-3 are also very similar having a mainly linear alpha helical shape, while CATH-2 contains a proline-induced hinge region in the middle of the peptide. This provides a kink in the three-dimensional structure of the peptide, which has been shown to be important for both antibacterial and immunomodulatory roles [10], [12]. CATH-1 and CATH-2 are mainly produced in bone marrow with lower expression levels in several other tissues. We recently showed that CATH-2 is present in heterophils and is released from these cells upon degranulation of these cells. The localization of CATH-3 has not been determined but is assumed to be similar to CATH-1 and -2 [13]. Contrary to this localization in immune cells, CATH-B1 is exclusively produced in the epithelial cells surrounding the M-cells in the bursa of Fabricius, suggesting that this peptide has a local role in forming a defense layer to protect the bursa from infection [7], although low levels of CATH-B1 RNA were also found in other tissues [14]. Despite the differences in localization, sequence and structure of the chicken cathelicidin subset, no significant functional differences between them have been identified.

**Table 1:** Amino acid sequence of mature chicken cathelicidins.

	Amino acid sequence	AA	Charge
CATH-1	RVKRVWPLVIRTVIAGYNLYRAIKKK	26	+8
CATH-2	RFGRFLRKIRRFRPKVTITIQGSARF	26	+9
CATH-3	RVKRFWPLVPVAINTVAAGINLYKAIRRK	29	+7
CATH-B1	PIRNWWIRIWEWLNGIRKRLRQRSPFYVRGHLNVTSTPQP	40	+7

To investigate the antimicrobial potential of chicken CATH-1-3, the minimal inhibitory concentration against a large set of clinically relevant bacterial species and strains was investigated. In addition, induction of resistance against cathelicidins was determined in three bacterial species.

## Materials and Methods

### Bacterial Isolates

In total 39 clinical bacterial isolates belonging to 25 Gram-negative and Gram-positive species were used in this study. The minimal inhibitory concentrations (MICs) of the isolates for different sets of antibiotics were determined according to the CSLI guidelines (Table S1) [15], [16]. The selection included methicillin-resistant *S. aureus*, vancomycin-resistant enterococci, ESBL-positive *Escherichia. coli* and carbapenemase-positive *Klebsiella pneumoniae*. MICs against the three isolates used in resistance development (see below) for the three chicken CATHs were determined using the same method.

### Peptides

The mature peptides of chicken CATH-1, -2 and -3 were synthesized by Caslo laboratory APS, Lyngby, Denmark. All three peptides were purified to >95% purity using HPLC, and mass spectrometry analysis indicated that the mass of the peptides were within 1 dalton of the theoretical value.

### Antimicrobial Activity Assays

#### -Colony count assays

Antimicrobial activity of the three chicken cathelicidins was tested against two MRSA, five ESBL-positive strains and a vancomycin-resistant *Enterococcus faecium*. Bacteria were maintained in Tryptic Soy Broth (TSB, Oxoid Limited, Hampshire, UK) at 37°C and grown to mid-logarithmic phase before testing. Colony count assays were performed to test the activity of CATHs as described previously [17]. In short, bacteria were pelleted and resuspended in 10 mM sodium phosphate buffer pH 7.0 containing 1/100 TSB and diluted to 2×10^6^ CFU/ml. A 25 µl aliquot of CATH peptide solution was mixed with 25 µl of bacterial culture and incubated for 3 h at 37°C. Subsequently, the cultures were diluted 50–5000 fold and spread plated on Tryptic Soy Agar plates and after 24 h at 37°C counted for surviving bacteria. The plates containing less than 10 colonies at the lowest sample dilution was defined as the minimal bactericidal concentration (MBC, >3 log reduction in CFU/ml).

#### -Spot-test

CATH-1, -2, and -3 were screened against 39 bacterial strains from different Gram-negative and Gram-positive species. The bacteria were cultivated overnight at 37°C, a suspension of bacteria (5×10^5^ CFU/ml) in distilled water was prepared and spread over Müller-Hinton (MH) agar plates. After the plates were dried, 20 µl of the CATH peptide solution (32 µM) was spotted. After overnight culture at 37°C the plates were examined for growth inhibition by measuring the diameter of the zones and noting the presence or absence of colonies within the spot area.

### Induction of CATH Resistance in *S. aureus* and *K. pneumoniae*

*S. aureus* S0385, *K. pneumoniae* 03C006, and *K. pneumoniae* NCTC13443 were cultured for 10 days in MH broth in the presence of CATH-1, -2, or -3, in a series of concentrations ranging from 0 to 40 µM. Daily, 50 µl samples displaying 80% growth compared with growth in MH broth only, were used to inoculate a new dilution series (0.5 ml). At day 10 broth dilution assays were performed to determine MIC values. Specifically, MICs were measured by sampling from the overnight cultures with highest peptide concentration that still showed detectable growth. These cultures were diluted till 2×10^6^ CFU/ml after which a 25 µl aliquot was incubated with 25 µl CATH (0–80 µM) for 3 h. Two-hundred µl MH broth was then added and incubation was continued for an additional 21 h. The concentration at which no visible bacterial growth was present was taken as the MIC value.

## Results

### Antimicrobial Activity Tests

#### -Colony count assays

**Figure 1:**
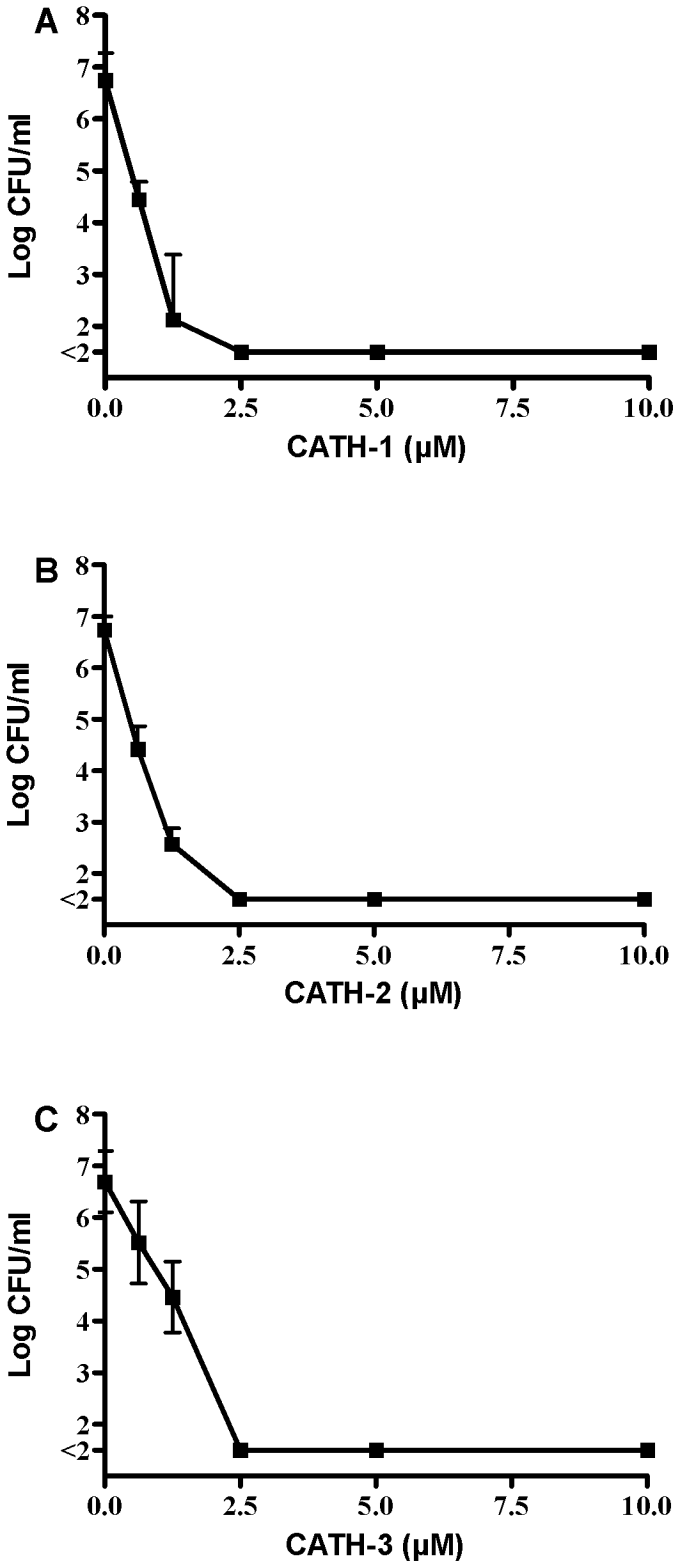
Antibacterial activity of CATH-1-3 against *E. coli* 38.34. *E. coli* (1×10^6^ CFU/ml) were incubated with CATH-1-3 for 3 h. Surviving bacteria were determined using colony count assays. All experiments were performed at least in triplicate. A: CATH-1; B: CATH-2; C: CATH-3.

MBCs for CATH-1-3 were determined using colony count assays against two MRSA, five ESBL-positive strains and a vancomycin-resistant *E. faecium*. The data for the activity of all three CATHs against ESBL-positive *E. coli* 38.34 is shown in Figure 1. At a concentration of 0.6 µM an approximately 2 log decrease in bacterial counts is observed for all three peptides. A higher concentration of peptide (2.5 µM) led to complete killing of all bacteria. MBC values using this method against the other strains tested are shown in Table 2 and indicate that no significant differences in MBC values were observed between the three peptides, and similar MBC values were observed for all bacteria tested. MIC values for the peptides determined by broth dilution assays in MH broth of the three isolates used in resistance development were in the same order of magnitude (0.63–1.25 µM).

**Table 2:** Antibacterial activity of chicken cathelicidins against multiresistant bacteria.

Bacterial strain		MBC (µM)		
	*	CATH-1	CATH-2	CATH-3
*Escherichia coli* 38.34	a	1.25–2.5	1.25–2.5	2.5
*Escherichia coli* 38.16	b	1.25–2.5	1.25–2.5	2.5–5
*Staphylococcus aureus* S0385	c	1.25	1.25–2.5	1.25–5
*Staphylococcus aureus* WKZ2	d	1.25–2.5	2.5	2.5
*Klebsiella pneumoniae* NCTC-13443	e	1.25–2.5	1.25–2.5	1.25–5
*Klebsiella pneumoniae* ATCC-BAA-1705	f	1.25–2.5	1.25–2.5	1.25–2.5
*Pseudomonas aeruginosa* VW178	g	0.6–1.25	1.25	1.25–2.5
*Enterococcus faecium* E155	h	0.6–1.25	1.25	1.25

* : a) CTX-M-1 ESBL positive from chicken, b) TEM-52 -ESBL positive from chicken, c) methicillin resistant, livestock-associated ST398, d) methicillin resistant, clinical isolate, e) NDM-1 carbapenemase positive, f) KPC carbapenemase positive, g) cystic fibrosis patients, h) vancomycin resistant. Bacteria (1×10^6^ CFU/ml) were incubated with CATH-1-3 for 3 h. Surviving bacteria were determined using colony count assays. All experiments were performed at least in triplicate.

#### -Spot-test

CATH-1, -2, -3 showed antibacterial activity against all 39 Gram-negative and Gram-positive bacterial strains belonging to 25 different species (Table 3). Peptide addition to a spread layer of Gram-positive bacteria resulted in clear inhibition zones for all bacteria tested. However, varying numbers of colonies were observed in the clear zone for most Gram-negative species. Exceptions were the *Acinetobacter* spp, *Stenotrophomonas maltophilia*, and one *K. pneumoniae* strain. The presence of colonies in the clear zone was strongest for CATH-1 where 13 of 18 tested Gram-negative strains (72%) showed colonies in the clear zone and lowest for CATH-3 where this effect was only present in 2 of the 18 (11%) strains. No difference was observed for ESBL- or carbapenemase-positive strains. For some bacteria, colonies from the clearance zone were grown overnight (in the absence of CATH) and used again for a spot test using the same peptide, to determine if total resistance was acquired. Without exception these experiments resulted in a similar clearance zone diameter and a comparable number of colonies in the clearance zone (data not shown) indicating the presence of heterogeneous resistance in these bacterial strains.

**Table 3:** Antimicrobial activity of chicken cathelicidins against a broad range of bacterial strains using spot-test.

			CATH-1		CATH-2		CATH-3	
Species	isolate nr.	*	zone Ø (mm)	colonies	zone Ø (mm)	colonies	zone Ø (mm)	colonies
*Enterococcus faecalis*	11E098		10	–	10	–	10	–
*Enterococcus faecalis*	15A374		9	–	9	–	9	–
*Enterococcus faecium*	E155	a	10	–	10	–	10	–
*Enterococcus faecium*	16D030		9	–	9	–	9	–
*Enterococcus faecium*	15A623		10	–	10	–	10	–
*Staphylococcus aureus*	S0385	b	10	–	11	–	11	–
*Staphylococcus aureus*	03A194		10	–	10	–	10	–
*Staphylococcus epidermidis*	08A1057		12	–	12	–	12	–
*Staphylococcus epidermidis*	08A1071		12	–	12	–	12	–
*Staphylococcus haemolyticus*	10A630		11	–	11	–	11	–
*Staphylococcus intermedius*	09D123		11	–	11	–	11	–
*Staphylococcus lugdunensis*	10A302		10	–	10	–	10	–
*Staphylococcus* species	19A337		11	–	11	–	11	–
*Streptococcus bovis*	12A090		10	–	8	–	8	–
*Streptococcus pyogenes*	05D015		12	–	11	–	11	–
*Streptococcus pyogenes*	23M092		11	–	11	–	11	–
*Streptococcus agalactiae*	05A396		10	–	10	–	10	–
*Streptococcus mitis*	01A162		10	–	10	–	10	–
*Streptococcus pneumonia*	14B186		11	–	11	–	11	–
*Streptococcus salivarius*	14A071		10	–	10	–	10	–
*Streptococcus sanguis*	08A557		11	–	11	–	11	–
*Acinetobacter baumannii*	06A330		10	–	10	–	10	–
*Acinetobacter calcoaceticus*	15A600		10	–	10	–	10	–
*Citrobacter diversus*	08A083		11		11	–	0	–
*Citrobacter freundii*	15C098		10	+	10	–	0	–
*Enterobacter aerogenes*	20A063		11	+	11	–	11	+++
*Escherichia coli*	01A280		11	+++	11	++	0	–
*Escherichia coli*	23E068		10	+	10	–	10	–
*Escherichia coli*	38.34	c	10	+++	11	–	0	–
*Escherichia coli*	38.16	d	0	+++	10	++	0	–
*Klebsiella pneumoniae*	03C006		10	–	10	–	0	–
*Klebsiella pneumoniae*	NCTC-13443	e	10	+++	11	–	0	–
*Klebsiella pneumoniae*	BAA-1705	f	11	++	10	+	0	–
*Pseudomonas aeruginosa*	VW178	g	10	+	10	+	0	–
*Pseudomonas aeruginosa*	04A191		10	+++	10	+++	0	–
*Pseudomonas aeruginosa*	13A066		10	+	10	+	10	++
*Salmonella enteritidis*	19A060		0	+++	10	–	0	–
*Salmonella typhimurium*	10A629		0	+++	10	++	0	–
*Stenotrophomonas maltophilia*	16C077		11	–	11	–	10	–

All strains were human isolates unless otherwise noted.

* : a) vancomycin resistant, b) methicillin resistant, livestock associated ST398, c) CTX-M-1 ESBL positive from chicken, d) TEM-52 -ESBL positive from chicken, e) NDM-1 carbapenemase positive, f) KPC carbapenemase positive, g) cystic fibrosis patients. Bacteria were cultivated overnight at 37°C, a suspension of bacteria (5×10^5 ^CFU/ml) in distilled water was prepared and spread over Müller-Hinton agar plates. After the plates were dried, 20 µl of a CATH solution (32 µM) was spotted, plates were cultured at 37°C and examined for growth inhibition by measuring the diameter of the zones and noting the presence or absence of colonies within the spot area. The table represents single or duplicate screening experiments.

### Induction of CATH Resistance in *S. aureus* and *K. pneumoniae*

**Figure 2:**
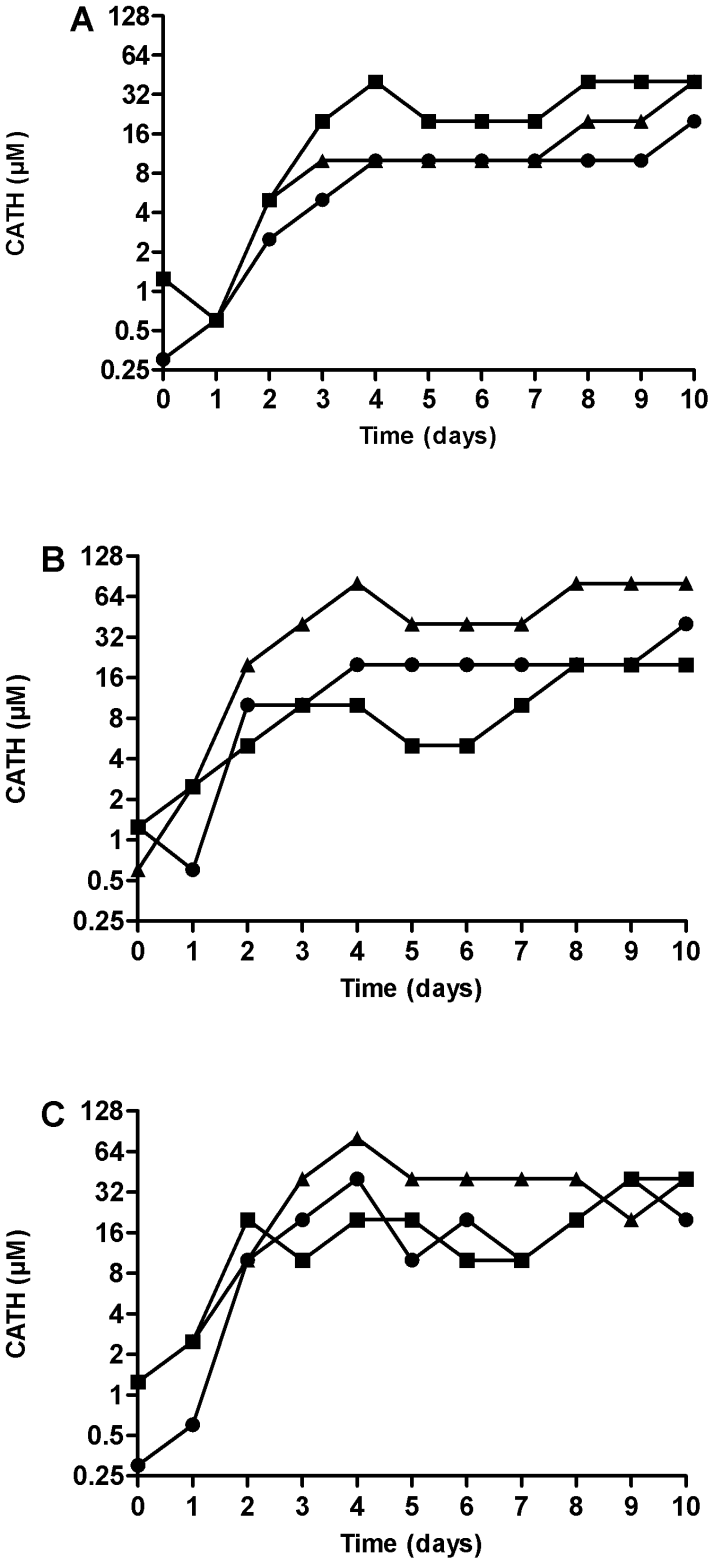
Induction of CATH-1, -2, and -3 resistance in *S. aureus* S0385 (panel A), *K. pneumoniae* NCTC-13443 (panel B) and *K. pneumoniae* 03C006 (panel C). Bacteria were grown o/n in the presence of 0–80 µM peptide. The sample containing the highest CATH concentration showing >80% bacterial growth compared to a control without CATH is shown. Subsequently, this bacterial culture was subcultured into new medium containing 0–80 µM CATH peptide. This procedure was repeated for 9 consecutive days. Circles: CATH-1; squares: CATH-2; triangles: CATH-3. Shown is the peptide tolerance (80% growth) over 10 days in a single induction experiment.

As a representative Gram-positive bacteria, *S. aureus* S0385 was used, whereas for Gram-negative bacteria two *K. pneumoniae* strains were selected: one strain that showed colonies in the clear zone in the spot test (*K. pneumoniae* NCTC13443) and one that did not show any colony growth in this zone (*K. pneumoniae* 03C006). All isolates reached maximum levels of reduced susceptibility within 4 days of the start of the experiment (Figure 2). No clear differences in the level of reduced susceptibility between the three CATHs were observed.

After day 10 of the induction with each CATH the MICs for each isolate from the dilution showing at least 80% growth were determined using broth dilution assays and colony count assays. Interestingly, the MIC values were much lower than the tolerated levels of CATHs during the resistance development experiment. In addition, although the MICs were higher for bacteria with reduced sensitivity than bacteria that did not have reduced sensitivity induced, the actual increase is still relatively small: on average 2–4 fold in the broth dilution assay (Table 4). These results were confirmed for *S. aureus* S0385 using colony count assays where even smaller, and in some cases no differences, were observed between the bacteria grown in the presence or absence of CATHs (data not shown).

**Table 4:** Antibacterial activity of chicken cathelicidins before and after induction of resistance.

	MIC (µM)					
	CATH-1		CATH-2		CATH-3	
Bacterial strain	Day 0	Day 10	Day 0	Day 10	Day 0	Day 10
*Staphylococcus aureus*S0385	0.3	1.25	1.25	5	1.25	1.25
*Klebsiella pneumoniae* *03C006*	0.6	2.5	1.25	10	1.25	1.25
*Klebsiella pneumonia*NCTC-13443	1.25	2.5	1.25	2.5	0.6	5

## Discussion

In this investigation we determined the antimicrobial activity of chicken CATHs 1–3 against ESBL-positive Enterobacteriaceae, MRSA, and other bacterial species. The fourth chicken cathelicidin CATH- B1 was left out of our studies due to its considerable bigger size and restricted localization, which lowers the potential of this peptide as alternative to antibiotics. Whether chicken CATHs are lytic to bacteria or possess a mode of action involving binding to intracellular targets is as of yet unknown. Interestingly, our results show no differences in MIC and MBC values for the three chicken CATHs tested, despite their considerable differences in amino acid sequence and structure, especially between CATH-2 and the other two CATH peptides. In addition, MBC values for all multiresistant strains tested were comparable to non-multiresistant strains [10]. This indicates that the antibacterial action of chicken CATHs is unrelated to the mechanism of action of classical antibiotics.

Remarkably, in the spot test heterogeneous resistance was present among the Gram-negative species tested, but absent among Gram-positive tested bacteria. Although it was not observed in this study, the phenomenon of heterogeneous resistance has been described for *Staphylococcus aureus*, where vancomycin and methicillin resistance can be heterogeneously present. In only approximately one in a million cells in a population, resistance is expressed. The exact mechanisms are still unknown, but for methicillin, next to the loss of a regulator protein, a chromosomal mutation appears to be necessary to obtain homogeneously expressed resistance [18]. An analogous phenomenon may occur here. Due to higher (or lower) than usual expression of resistance, e.g. due to a regulator, in only a few cells in a population, resistant colonies survive on a plate. In induction experiments these cells are increasingly selected and a mutation may further enhance the resistance level. When the culture is no longer exposed, revertants will take over due to a fitness advantage and resistance levels drop to wild-type values.

In the induction experiments with *K. pneumoniae* and *S. aureus*, reduced sensitivity to all three CATHs tested,was obtained within a few days. The mechanism(s) explaining these observations are not known because resistance to cationic antimicrobial peptides, including cathelicidins, is only partially understood. Different peptides and different bacterial species or groups appear to have different resistance mechanisms [19]. In *S. aureus* at least five different mechanisms exist including production of proteases and HDP inactivating proteins, alteration of membrane fluidity and membrane charge and expression of multidrug pumps [20]. Several of these mechanisms have also been shown to be induced in the presence of HDPs through the activation of bacterial two- and three-component systems. In Gram-negative bacteria similar mechanisms exist, e.g., *phoPQ* regulated genes [21]–[23]. An efflux pump has been implicated in *Neisseria gonorrhoeae* [24], a *phoPQ* regulated protease in *Salmonella enterica* serovar Typhimurium [21], and a protease in enterohemorrhagic *E. coli* [25]. However, these mechanisms mostly seem to involve an intrinsic resistance to cathelicidins, because knock-outs of the proposed genes result in reduced resistance compared to wild-type.

In the 10 day resistance induction experiment, overnight growth was used as read out parameter but this has only limited value since a very small number of surviving bacteria could potentially grow out to a full density overnight culture. Indeed growth curves of bacteria in the presence of sub-MIC concentration of CATH show that the peptide increases the time till exponential growth phase is reached (data not shown). Obviously, in vivo this delay could be enough for other immune factors to effectively eradicate the remaining bacteria. In addition, the actual MIC determined by colony count assays and broth dilution assays were much lower than the concentration where overnight growth was observed, and only slightly higher compared to non-induced bacteria. The larger volumes used during the induction experiment compared to broth dilution assays might partially explain this difference. If heterogeneous resistance is present, the chance of having resistant bacteria is higher at larger volumes. More importantly, the number of bacteria transferred to the next generation (∼10^7^ CFU/ml) is 10 fold higher than used in the broth dilution assays, again increasing chances of transferring resistant bacteria. Finally, in experiments in our group using fluorescently labeled peptides, it was observed that peptides instantly localize to microbial membranes and that the peptide was often heterogeneously distributed among cells. This heterogeneous distribution would lead to bacterial cells receiving sub-MIC concentrations of peptide, enabling them to eventually grow out to a proper overnight culture. Overall, CATH resistance as determined by MIC values only increases slightly and this small increase is achieved within a few days and does not seem to develop further upon prolonged incubation periods. This indicates that, at least in our experimental set-up, no major resistance mechanisms leading to loss of susceptibility towards CATHs are induced, contrary to the resistance development described for more classic antibiotics.

In summary, our experiments show an antimicrobial activity of CATH-1, -2, and -3 against both Gram-negative and Gram-positive bacteria, independent of the presence of resistance mechanism towards classic antibiotics. No clear differences in activity were observed between the three CATHs. Heterogeneous resistance was noted in Gram-negative species in spot assays, but induction of resistance towards chicken CATHs was low and leveled off after 3–4 days indicating that development of major resistance is unlikely to occur.

## Supporting Information

Table S1 Susceptibility of bacterial strains used in this study for a large set of antibiotics. MICs were determined according to CSLI guidelines.
(XLSX)
